# Assessment of low concentration wastewater treatment operations with dewatered alum sludge-based sequencing batch constructed wetland system

**DOI:** 10.1038/s41598-017-17783-3

**Published:** 2017-12-13

**Authors:** Wei Kang, Hongxiang Chai, Yu Xiang, Wei Chen, Zhiyu Shao, Qiang He

**Affiliations:** 1 Key Laboratory of Three Gorges Reservoir Region’s Eco-Environment, Ministry of Education, Chongqing University, Chongqing, 400045 P.R. China; 2 National Centre for International Research of Low-carbon and Green Buildings, Chongqing University, Chongqing, 400045 P.R. China

## Abstract

Competition of volatile fatty acids between anoxic denitrification and anaerobic phosphorus release is prominent. Therefore, low concentration wastewater has restricted effects on nitrogen and phosphorus removal. The purpose of this study is to treat dormitory sewage with a biochemical oxygen demand (BOD) ranging from 50 to 150 mg/L using dewatered alum sludge-based sequencing batch constructed wetland system. Vegetation in the wetland system was chosen to be *Phragmites australis*. Three parallel cases were carried out to assess impacts due to different hydraulic retention time (HRT) and artificial aeration. The results showed that this system is effective in removing total nitrogen (TN), ammonia nitrogen (NH_3_-N) and total phosphorus (TP) under different HRT. However, nitrous oxide (N_2_O) emission poses to be the greatest challenge in the high HRT cases. Artificial aeration could reduce N_2_O emission but is associated with high operational cost. Results indicate that dewatered alum sludge-based sequencing batch constructed wetland system is a promising bio-measure in the treatment of low concentration wastewater.

## Introduction

Low concentration wastewater refers to the type of sewage water that has a chemical oxygen demand (COD) concentration lower than 1000 mg/L or a biochemical oxygen demand (BOD) concentration lower than 500 mg/L, which is mainly composed of municipal sewage and diluted industrial wastewater^1^. Due to the lack of carbon source, low concentration wastewater cannot provide enough nutrients for microorganisms, which affects anaerobic phosphorus release, anoxic denitrification and aerobic heterotrophic bacteria metabolism. Treatment technologies of low concentration domestic sewage are based on traditional aerobic treatment, including activated sludge, contact oxidation and drop filter aerobic process. However, these processes involve high energy consumption, investment and operating costs. At the same time, it was believed that the performance of anaerobic treatment is significantly affected by substrate limitation. Also, the anaerobic oxidation rate is very slow at low concentration, and has little effects on the removal of organic COD in wastewater^2,3^.

Constructed wetland has been increasingly used to reduce excessive nutrient loading caused by human activities for many advantages, including low cost, easy to operate and low maintenance^4,5^. Based on the water flow regime and the type of macrophytic growth, constructed wetland could be classified into three groups: free water surface flow constructed wetland, subsurface flow constructed wetland, and hybrid systems^6^. Two types of small-scale surface flow constructed wetland, including unplanted and planted with various macrophytes, were used by Lin *et al*. to continuously receive nitrate-contaminated groundwater. The efficiency ranged from 70% to 99% for planted wetland cell and was abut 50% for unplanted wetland cell^7^. Chang *et al*. adopted integrated vertical flow constructed wetland system in the treatment of nitrate-laden wastewater, and the mean removal efficiency of nitrate and total nitrogen (TN) reached 56.2% and 55.1% under a relatively low influent COD: N ratio of 1.67^8^. By integrating three stages of vertical-flow and horizontal-flow constructed wetland, the series-parallel mode had the highest TN removal efficiency (45.2%) at an hydraulic retention time (HRT) of 3 h and the highest total phosphorus (TP) removal efficiency (74.6%) at an HRT of 5 h^9^.

In order to enhance the purification ability of conventional constructed wetland, many approaches have been studied in recent years. Intermittent feeding strategy has been proved to be an effective method. The intermittent operation turned constructed wetland into a biological reactor with anoxic, anaerobic and aerobic conditions, which greatly enhanced the removal of ammonia nitrogen (NH_3_-N), COD and TP^10–12^. However, the influence mechanism of intermittent operation on TN removal was not clear. Jia *et al*. adopted vertical flow constructed wetlands to study the influences of intermittent operation and different length of drying time on contaminant removal. In their study, it was concluded that the intermittent operation could promote the nitrification but the denitrification was inhibited, mainly due to the higher dissolved oxygen (DO) concentration and the lack of carbon source^13^. Nevertheless, Foladori *et al*. adopted vertical subsurface-flow constructed wetlands to treat high hydraulic and organic loads, and proposed that the aerated and recirculated wetland resulted in a higher TN removal due to simultaneous nitrification/denitrification^14^.

However, the intermittent operation could promote the emission of nitrous oxide (N_2_O), and the amount of N_2_O emission from the subsurface flow constructed wetlands with intermittent operation was about 5 times higher than that with continuous operation^15^. The constructed wetland systems with certain types of vegetation even raise the N_2_O released much higher. The study revealed that Zizania latifolia had a larger contribution to N_2_O emission, comparing with *Phragmites australis* and *Typha latifolia* ^16,17^. Meanwhile, Wu *et al*. studied the N_2_O fluxes of *Phragmites australis*, *Scirpus validus*, *Iris pseudacorus*, *Zizania latifolia*, *Lythrum salicaria* and *Typha latifolia*, and found that the higher risk of N_2_O emissions was observed in constructed wetland planted with *Typha latifolia* and *Scirpus validus*, while the lowest N_2_O emission was in *Phragmites australis* systems^18^. Therefore, the choice of aquatic plant can be used as an effective way to reduce N_2_O emission in constructed wetlands.

Water treatment sludge is a by-product of coagulation, flocculation and other traditional water treatment processes. Sludge from water treatment plant could effectively improve soil structure, increase water retention ability, and enhance supply capacity of nutrients needed by plants^19–21^. Meanwhile, there was no evidence that aluminium toxicity would be a problem if alum sludge was used as plant growth media^22^. Zhao *et al*. and Yang *et al*. integrated the alum sludge, a by-product of drinking water treatment plant when aluminium salt is added as a coagulant, into a constructed wetland system for P-rich wastewater treatment. The results demonstrated that alum sludge can secure the P removal, and active bacteria attached growth on the alum sludge can effectively remove organic matter and NH_3_-N^23,24^. In addition, there are studies indicating that P-adsorption capacity could be reduced with the increase of alum sludge particle sizes, and P recovery after the slum sludge reuse is feasible from a technical point of view^25^. However, the TP removal mechanism by alum sludge in the above study was not explicitly revealed.

In this study, a dewatered alum sludge-based sequencing batch constructed wetland system planted with *Phragmites australis* was designed to treat low concentration wastewater. Because physical, chemical, and biological wetland treatment processes are all a function of time, increasing HRT will improve the treatment effect^26–28^. In the limiting DO conditions, ammonia-oxidizing bacteria can use nitrite nitrogen as the electron acceptor, which could increase the release of N_2_O^29,30^. By studying the influence of HRT on the removal of TN, NH_3_-N and TP, the influence mechanism of intermittent operation on TN removal and the TP removal pathway by alum sludge were revealed. Furthermore, the choice of *Phragmites australis*, HRT and artificial aeration could effectively alleviate the influence of intermittent operation on N_2_O emission.

## Materials and Methods

### Experiment setting

**Figure 1:**
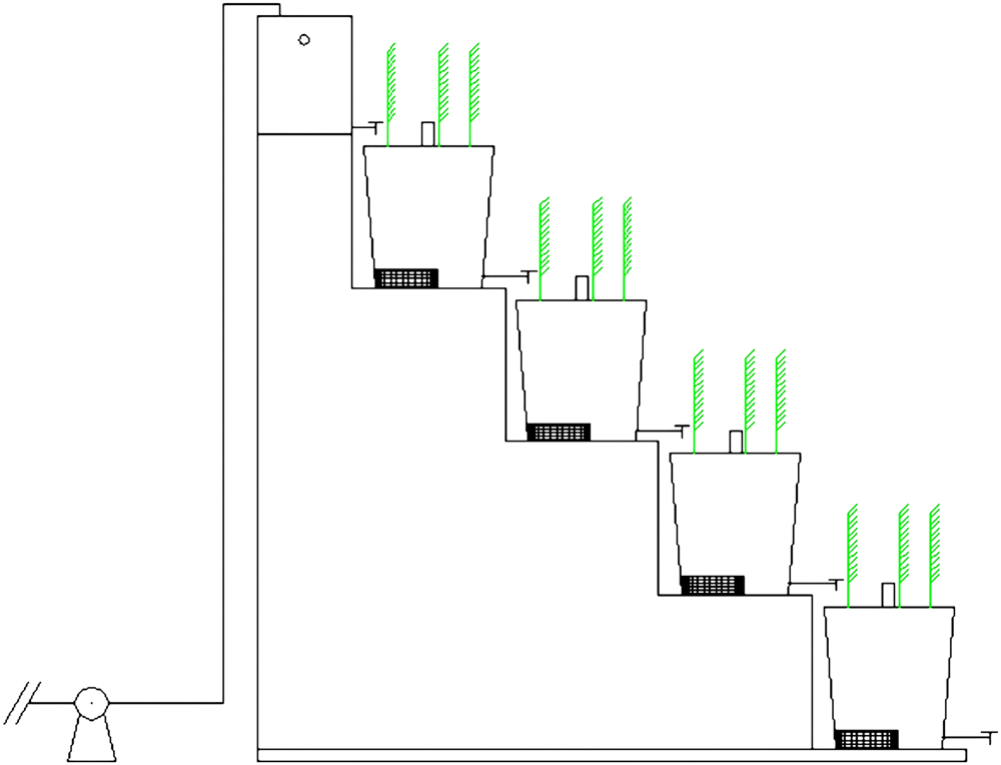
Schematic diagram of dewatered alum sludge-based sequencing batch constructed wetland system.

The schematic diagram of dewatered alum sludge-based sequencing batch constructed wetland system in this study was shown in Fig. 1.

The dewatered alum sludge-based sequencing batch constructed wetland system was located at Chongqing University. Considering the hilly terrain and significant elevation variance in the city of Chongqing, this system could utilize the natural elevation drops to create a nature aeration effect. The system in this study was composed of top water tank and four constructed wetlands inseries. Young *Phragmites australis* was planted on top of each constructed wetland. A perforated tube was placed at the center of each constructed wetland for emplacing the probe of DO monitor and pH instrument. Artificial aeration was achieved with a diaphragm air pump that diffused air continuously near the bottom of the inlet of each constructed wetland.

The effective volume of top water tank was 64 L, with size of L × B × H = 40 × 40 × 40 cm. The size of four constructed wetlands inseries was identical, and effective volume for each constructed wetland was 80 L. Each individual constructed wetland was filled with a 25 mm-diameter gravel layer as the distribution layer up to 5 cm deep from the top. Then the gravel layer was followed by 50 cm of dewatered alum sludge as the main substrate layer. Then 5 cm deep of 10 mm-diameter gravel was filled at the bottom and served as a supporting layer.

### Dewatered alum sludge and wastewater

The experimental dewatered alum sludge was collected from the industrial filter press of the sludge dewatering unit in a local water treatment plant in Chongqing, China, where aluminium sulphate was used as coagulant. Thereafter, the dewatered alum sludge was air-dried at room temperature for 10 days. The air-dried dewatered alum sludge was then ground and sieved to prepare the sludge for batch P-adsorption test. The main physicochemical properties of dewatered alum sludge were shown in Table 1.

**Table 1:** Main physicochemical properties of dewatered alum sludge.

Index	TP	Al_ox_	Fe_ox_	Al	Fe	Ca	Mg
Concentration(g/kg)	0.48	56.65	8.56	58.70	9.25	5.36	5.45

The experimental wastewater used in this study was collected from student dormitory at Chongqing University. Taking into account the unique life regulation of college students, the wastewater quality of this experiment had a wide variation range, which had a concentration of 49.8–150.3 mg/L (BOD), 60.2–140.4 mg/L (TN), 48.8–96.3 mg/L (NH_3_-N), 3.0–5.3 mg/L (TP) and 7.1–8.3 (pH). During this experiment, dormitory wastewater was diluted with tap water if necessary and was used as influent to constructed wetland system.

### Experimental procedure

At the beginning, the pump drew dormitory wastewater to the top water tank. Under the action of gravity, wastewater flowed into the constructed wetland in series through a perforated tube at the bottom of each individual wetland. Then the constructed wetland system entered reaction stage after all valves were closed. At the end of reaction stage, the last level constructed wetland began to drain. The upper level constructed wetland began to drain after the last level constructed wetland was evacuated. This drainage process continued until influent of the first level constructed wetland was completed.

All experiments in this study were carried out in three parallel cases, and the temperature during operation was remained to be 25–30 °C. On the one hand, under the precondition of non artificial aeration, the influence of different HRT (4, 8, 16, 24 h) on the removal of TN, NH_3_-N and TP was studied in this study. On the other hand, the influence of different HRT (4, 8, 16, 24 h) and different artificial aeration intensity (1, 2, 3, 4 m^3^/m^2^/h) on the N_2_O emission were investigated in this study.

### Samples and analyses

During the constructed wetland system operation period, samples of influent and effluent from each level constructed wetland were collected periodically and analyzed. Wastewater temperature was measured by a portable probe. TN was measured by the persulphate digestion and oxidation-double wavelength method. NH_3_-N was measured by Nessler’s reagent colorimetry and determined using Hach DR6000. TP was measured colorimetrically by the persulfate digestion-molybdophosphate reaction method. Removal efficiencies were obtained by calculating the percentages of pollutant removal from the influent concentrations^31,32^.

The N_2_O concentration was determined using the gas chromatography, and the N_2_O emission flux was calculated by the following formula described by Wu *et al*.^33^:

$${\rm{J}}=\frac{{\rm{dc}}}{{\rm{dt}}}\cdot \frac{{\rm{M}}}{{{\rm{V}}}_{0}}\cdot \frac{{\rm{P}}}{{{\rm{P}}}_{0}}\cdot \frac{{\rm{T}}}{{{\rm{T}}}_{0}}\cdot {\rm{H}}\cdot 1000$$

where J represents emission flux; dc/dt represents slope of gas concentration plot vs. time plot; M represents mole mass of N_2_O; P represents atmosphere pressure; T represents absolute temperature; V_0_, P_0_, T_0_ represent volume, atmosphere pressure and temperature under standard condition, respectively; and H represents chamber height above water surface.

## Results and Discussion

### Pollutant removal efficiency

**Figure 2:**
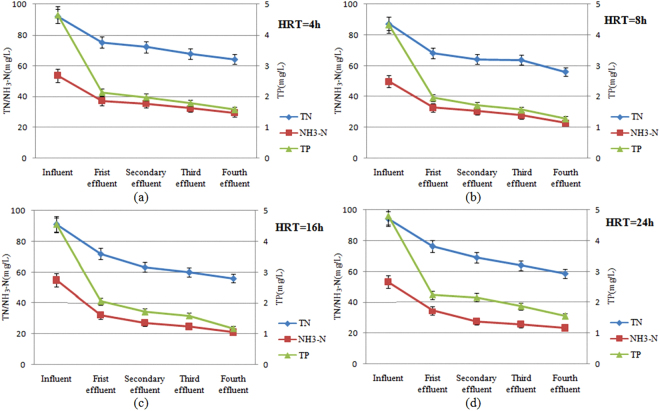
Influence of HRT on the removal rate of TN, NH_3_-N and TP.

Expeimental results of TN, NH_3_-N and TP removal rate under different HRT were shown in Fig. 2:

Figure 2 showed that, majority TN, NH_3_-N and TP in the influent was removed in the first level constructed wetland. It also could be observed that, the increase of HRT would prolong the contact time between some microbes and organic matter in wastewater and hence improved the pollutant removal efficiency. However, when the HRT increased from 16 h to 24 h, the removal rate of TN, NH_3_-N and TP decreased from 38.33%, 61.87% and 74.29% to 37.68%, 56.30% and 67.78%, respectively. This was in accordance with the theory in which the exorbitant HRT would easily induce the trapped organic matter to be dissolved into wastewater.

The analysis showed that, the shorter HRT could cause ammonia in the wastewater to be insufficient for contacting with nitrifying bacteria due to the slow growth of autotrophic bacteria. At the same time, the shorter HRT results in a higher inflow, which leads to the reproduction rate of nitrifying bacteria lower than loss rate. In addition, with the increase of HRT, the adsorption of dewatered alum sludge on NH_3_-N in every level constructed wetland all was close to a saturation level. Hence, prolonging HRT was not an effective way to increase NH_3_-N removal rate^34,35^.

In the meantime, in the case of the shorter HRT, the drop-aeration in constructed wetland system could lead to a high DO concentration while a higher influent flow caused by the shorter HRT also could lead to a large loss of nitrifying bacteria. In the case of the longer HRT, the expansion of anaerobic zone in the constructed wetland system would decrease the activity of aerobic microbe, and the heavy consumption of organic carbon source would also reduce the activity of denitrifying bacteria. This agrees with the theory that intermittent operation could promote the nitrification but the denitrification was inhabited. Therefore, the intermittent operation always inhibited the removal of TN regardless of the value of HRT^36,37^.

Previous studies have shown that the shorter HRT could cause phosphate in the wastewater to be insufficient for contacting with phosphate accumulating organisms, and the longer HRT could reduce the activity of phosphate accumulating organisms due to the competition between this organisms and other microorganisms^28,38^. In this study, the experimental data indicated that the longer HRT lead to a higher TP removal efficiency. Therefore, the removal of TP in this study is not mainly dependent on the role of phosphate accumulating organisms. During the experiment process, the decrease of TP concentration was accompanied with an increase in pH values and nitrate concentration. This suggests that phosphate ions replaced -OH on the surface of dewatered alum sludge. It could be inferred that ligand exchange might be the dominating pathway for phosphate removal^39,40^.

### N_2_O emission flux

**Figure 3:**
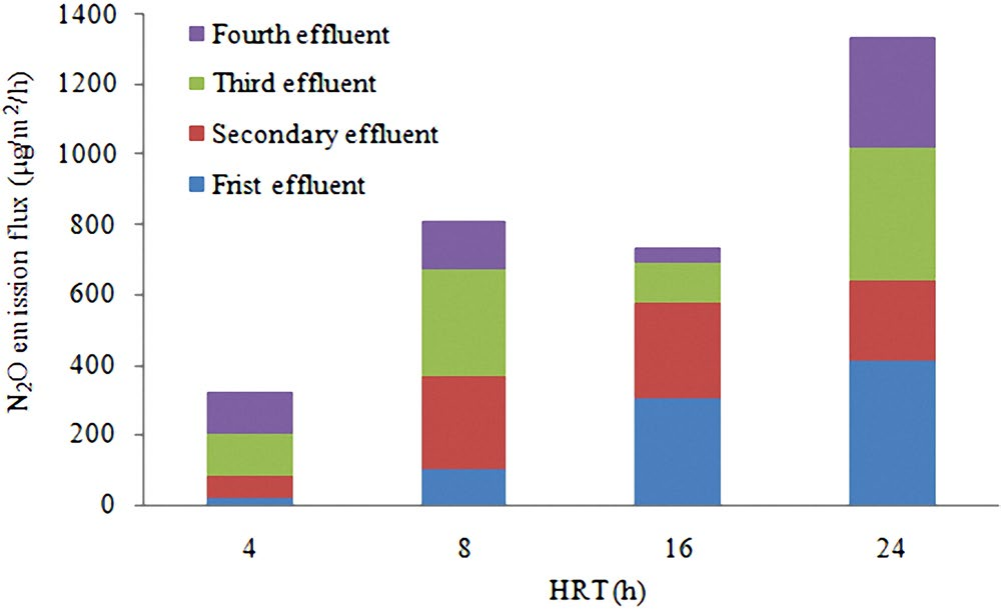
Influence of HRT on N_2_O emission flux.

The results of the test about HRT on N_2_O emission flux during non artificial aeration phase were shown in Fig. 3.

The influence of HRT on N_2_O emission flux was significant; this could be concluded from Fig. 3. With the increase of HRT, N_2_O emission flux in different level constructed wetland all basically increased. In the meantime, different level constructed wetland in the same HRT had different N_2_O emission flux. However, the experimental data shown that N_2_O emission flux in HRT of 16 h could not meet the change law described above.

The analysis showed that, DO concentration in constructed wetland system decreased with the increase of HRT. Hence it reduced the activity of nitrous oxide reductase and eventually promoted N_2_O emission. Furthermore, the lower DO concentration could limit the oxidation of nitrite, which may promote N_2_O emission under the premise of the existence of nitrite reductase^41,42^. In addition, intermittent operation could cause more oxidizing conditions, which lead to N_2_O emission when DO was present in low amounts. However, the experimental data shown that N_2_O emission flux in HRT of 16 h could not meet the change law described above, which may be related to the removal rate of TN and NH_3_-N.

**Figure 4:**
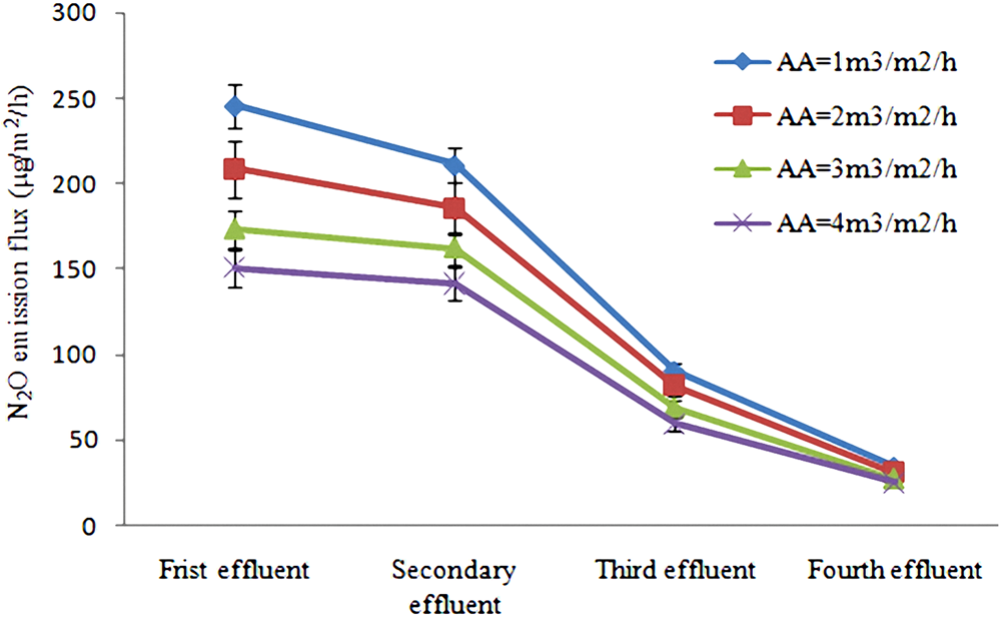
Influence of artificial aeration on N_2_O emission flux.

It could be concluded from Figs 2 and 3 that, when HRT was 16 h, the removal rate of TN, NH_3_-N and TP all reached the maximum value, and N_2_O emission flux reached the small value. Therefore, it is believed that 16 h is the optimal HRT for this constructed wetland system. In order to solve the global warming problem caused by this constructed wetland system, artificial aeration could be as an effective way to reduce the N_2_O emission, and the results of the test about artificial aeration on N_2_O emission flux during 16 h of HRT were shown in Fig. 4.

The analysis of Fig. 4 showed that, the enhancement of artificial aeration could increase DO concentration, which in turn improves the activity of nitrous oxide reductase and eventually reduces N_2_O emission. However, the decreased degree of artificial aeration on DO concentration is limited, which agrees well with the theory that exorbitant DO concentration has a strong toxicity to nitrous oxide reductase^43,44^.

**Figure 5:**
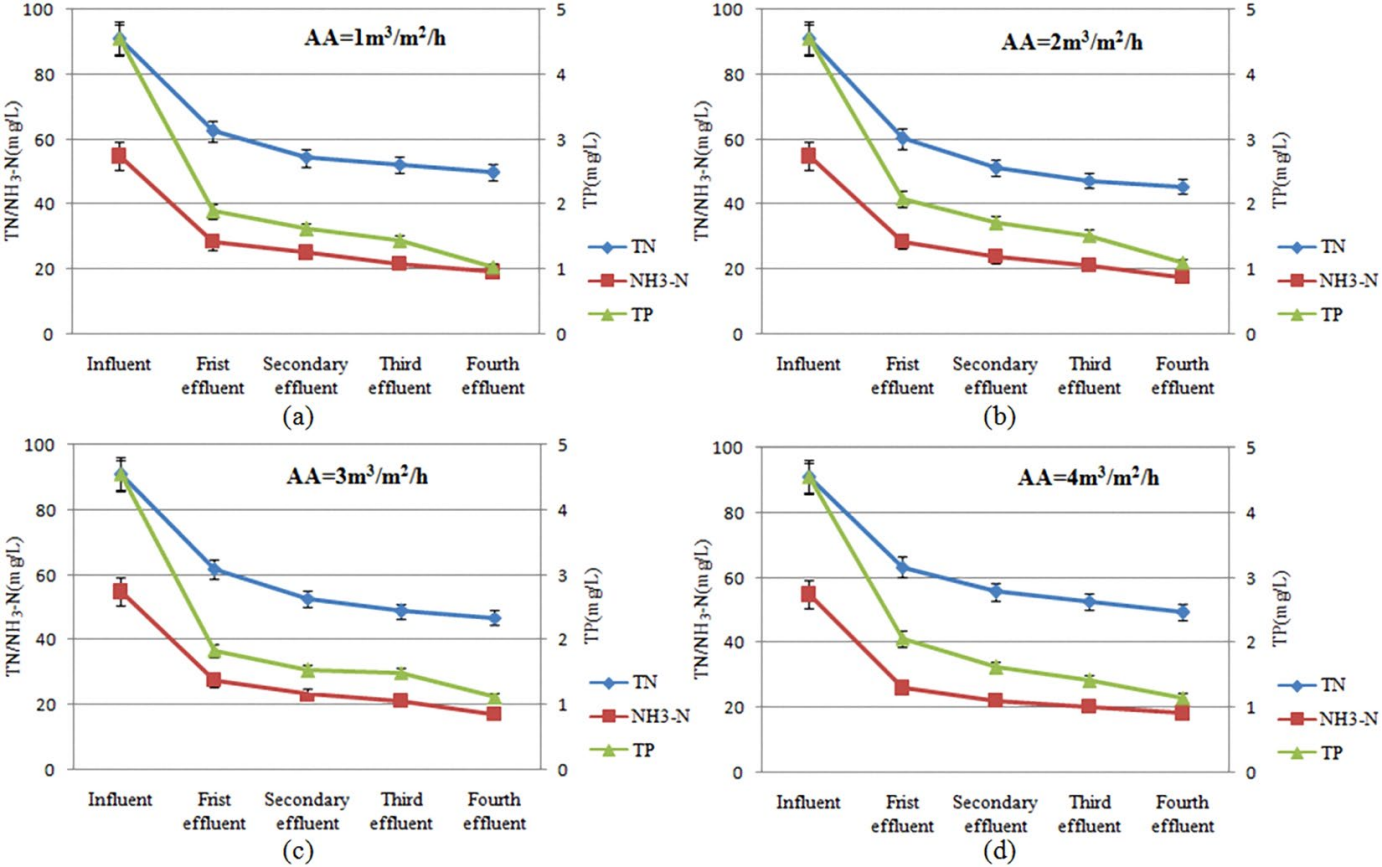
Influence of artificial aeration on the removal rate of TN, NH_3_-N and TP.

In the meantime, the results of the studies of removal of TN, NH_3_-N and TP under different artificial aeration at a HRT of 16 h were shown in Fig. 5.

The analysis showed that, artificial aeration could enhance oxidation ratio of NH_3_-N, and improve mass transfer efficiency of TN and TP^45,46^. However, exorbitant DO concentration would inhibit the denitrification process and lead to the desorption of TP adsorbed on the substrate surface^43,44^. In addition, the enhancement of artificial aeration is associated with higher operational cost^47^. As a result, the selection of artificial aeration could be balanced by N_2_O emission standard and economic feasibility.

## Conclusions

The dewatered alum sludge-based sequencing batch constructed wetland planted with *Phragmites australis* was used to treat dormitory sewage. The results showed that intermittent operation always inhibited the removal of TN regardless of the value of HRT, and ligand exchange was the dominating pathway for phosphate removal. In addition, the selection of artificial aeration and HRT could be balanced by N_2_O emission standard and economic feasibility.

### Data availability

No datasets were generated or analysed during the current study.

## References

[1] Shin HS, Park MG, Jung JY (2001). Nutrient removal processes for low strength wastewater. Environ. Technol..

[2] Fan J, Tao T, Zhang J, You GL (2009). Performance evaluation of a modified anaerobic/anoxic/oxic (A^2^/O) process treating low strength wastewater. Desalination.

[3] Zhang HM (2011). Aerobic granulation with low strength wastewater at low aeration rate in A/O/A SBR reactor. Enzyme Microb. Tech..

[4] Vymazal, J. (Ed.) Natural and constructed wetlands: Nutrients, metals and management. Backhuys Publishers, Leiden (2005).

[5] Inamori R (2007). Investigating CH_4_ and N_2_O emissions from eco-engineering wastewater treatment processes using constructed wetland microcosms. Process Biochem..

[6] Zhang DQ (2015). Application of constructed wetlands for wastewater treatment in tropical and subtropical regions (2000–2013). J. Environ. Sci-CHINA.

[7] Lin YF, Jing SR, Lee DY, Chang YF, Shih KC (2007). Nitrate removal and denitrification affected by soil characteristics in nitrate treatment wetlands. J. Environ. Sci. Heal A.

[8] Chang JJ, Wu SQ, Dai YR, Liang W, Wu ZB (2013). Nitrogen removal from nitrate-laden wastewater by integrated vertical-flow constructed wetland systems. Ecol. Eng..

[9] Ren LJ (2015). Effects of connection mode and hydraulic retention time on wastewater pollutants removal in constructed wetland microcosms. Clean-Soil Air Water.

[10] Chan SY, Tsang YF, Cui LH, Chua H (2008). Domestic wastewater treatment using batch-fed constructed wetland and predictive model development for NH_3_-N removal. Process Biochem.

[11] Chan SY, Tsang YF, Chua H, Sin SN, Cui LH (2008). Performance study of vegetated sequencing batch coal slag bed treating domestic wastewater in suburban area. Bioresource Technol..

[12] Caselles-Osorio A, Garcia J (2007). Impact of different feeding strategies and plant presence on the performance of shallow horizontal subsurface-flow constructed wetlands. Sci. Total Environ..

[13] Jia WL, Zhang JA, Wu JA, Xie HJ, Zhang B (2010). Effect of intermittent operation on contaminant removal and plant growth in vertical flow constructed wetlands: A microcosm experiment. Desalination.

[14] Foladori P, Ruaben J, Ortigara ARC (2013). Recirculation or artificial aeration in vertical flow constructed wetlands: A comparative study for treating high load wastewater. Bioresource Technol..

[15] Jia WL (2011). Nitrous oxide emissions from surface flow and subsurface flow constructed wetland microcosms: Effect of feeding strategies. Ecol. Eng..

[16] Wang YH (2008). Nitrous oxide emission from polyculture constructed wetlands: Effect of plant species. Environ. Pollut..

[17] Inamori R (2008). Seasonal effect on N_2_O formation in nitrification in constructed wetlands. Chemosphere.

[18] Wu, J., Zhang, H., Jia, W. L., Xie, H. J. & Wu, H. M. Effects of plant species on nitrous oxide emission and microbial community structure diversity in constructed wetlands. 2009 International Conference on Environmental Science and Information Application Technology 160–163 (2009).

[19] Rengasamy P, Oades JM, Hancock TW (1980). Improvement of soil structure and plant-growth by addition of alum sludge. Commun. Soil Sci. Plan..

[20] Kim JG, Lee SS, Moon HS, Kang IM (2002). Land application of alum sludge from water purification plant to to acid mineral soil treated with acidic water. Soil Sci. Plant Nutr..

[21] Park SG, Yahata H, Saeki K, Kurosawa K, Kim YJ (2009). Physical properties of water treatment residue and their effects on plant growth as a substitute soil. J. Fac. Agr. Kyushu U..

[22] Skene TM, Oades JM, Kilmore G (1995). Water treatment sludge: A potential plant growth medium. Soil Use Manage..

[23] Zhao YQ, Babatunde AO, Zhao XH, Li WC (2009). Development of alum sludge-based constructed wetland: An innovative and cost effective system for wastewater treatment. J. Environ. Sci. Heal A.

[24] Yang Y (2011). A promising approach of reject water treatment using a tidal flow constructed wetland system employing alum sludge as main substrate. Water Sci. Technol..

[25] Zhao XH (2015). Key issues to consider when using alum sludge as substrate in constructed wetland. Water Sci. Technol..

[26] Conn RM, Fiedler FR (2006). Increasing hydraulic residence time in constructed stormwater treatment wetlands with designed bottom topography. Water Environ. Res..

[27] Zhang CB (2012). Effects of plant diversity and hydraulic retention time on pollutant removals in vertical flow constructed wetland mesocosms. Ecol. Eng..

[28] Wang H, Huang CC, Ge Y, Wu JZ, Chang J (2014). The performance of species mixtures in nitrogen and phosphorus removal at different hydraulic retention times. Pol. J. Environ. Stud..

[29] Otte S, Grobben NG, Robertson LA, Jetten MSM, Kuenen JG (1996). Nitrous oxide production by Alcaligenes faecalis under transient and dynamic aerobic and anaerobic conditions. Appl. Environ. Microb..

[30] Platzen L, Koch-Koerfges A, Weil B, Brocker M, Bott M (2014). Role of flavohaemoprotein Hmp and nitrate reductase NarGHJI of Corynebacterium glutamicum for coping with nitrite and nitrosative stress. Fems Microbiol. Lett..

[31] APHA. Standard Methods for the Examination for Water and Wastewater, 22nd ed (2012).

[32] Li Y (2011). Integration of algae cultivation as biodiesel production feedstock with municipal wastewater treatment: strains screening and significance evaluation of environmental factors. Bioresource Technol..

[33] Wu J (2009). Impact of COD/N ratio on nitrous oxide emission from microcosm wetlands and their performance in removing nitrogen from wastewater. Bioresource Technol..

[34] Wang ZC (2015). Effect of hydraulic retention time on performance of an anoxic-aerobic sequencing batch reactor treating saline wastewater. Int. J. Environ. Sci. Te..

[35] Li, L., Yao, J., Fang, X. Y., Huang, Y. X. & Mu, Y. Electrolytic ammonia removal and current efficiency by a vermiculite-packed electrochemical reactor. *Sci Rep-UK* **7** (2017). 10.1038/srep41030 PMC5244476 28102340

[36] Nogueira R, Melo LF, Purkhold U, Wuertz S, Wagner M (2002). Nitrifying and heterotrophic population dynamics in biofilm reactors: Effects of hydraulic retention time and the presence of organic carbon. Water Res..

[37] Li D, Lv YF, Cao MZ, Zeng HP, Zhang J (2016). Optimized hydraulic retention time for phosphorus and COD removal from synthetic domestic sewage with granules in a continuous-flow reactor. Bioresource Technol..

[38] Tang XQ, Huang SL, Fciwem MS (2009). Comparison of phosphorus removal between vertical subsurface flow constructed wetlands with different substrates. Water Environ. J..

[39] Huang SH, Chiswell B (2000). Phosphate removal from wastewater using spent alum sludge. Water Sci. Technol..

[40] Yang Y (2006). Characteristics and mechanisms of phosphate adsorption on dewatered alum sludge. Sep. Purif. Technol..

[41] Frison N (2015). Mitigating off-gas emissions in the biological nitrogen removal via nitrite process treating anaerobic effluents. J. Clean Prod..

[42] Morozkina EV, Kurakov AV (2007). Dissimilatory nitrate reduction in fungi under conditions of hypoxia and anoxia: a review. Prikladnaia biokhimiia i mikrobiologiia.

[43] Maltais-Landry G, Maranger R, Brisson J, Chazarenc F (2009). Greenhouse gas production and efficiency of planted and artificially aerated constructed wetlands. Environ. Pollut..

[44] Maltais-Landry G, Maranger R, Brisson J (2009). Effect of artificial aeration and macrophyte species on nitrogen cycling and gas flux in constructed wetlands. Ecol. Eng..

[45] Vymazal J (2007). Removal of nutrients in various types of constructed wetlands. Sci. Total Environ..

[46] Tang XQ, Huang SL, Scholz M (2008). Nutrient removal in wetlands during intermittent artificial aeration. Environ. Eng. Sci..

[47] Verrecht B, Maere T, Benedetti L, Nopens I, Judd S (2010). Model-based energy optimisation of a small-scale decentralised membrane bioreactor for urban reuse. Water Res..

